# Less water, same buds: moderate drought as a water-saving strategy for indoor cannabis

**DOI:** 10.3389/fpls.2026.1930650

**Published:** 2026-09-03

**Authors:** Danilo Crispim Massuela, Sebastian Munz, Jens Hartung, Alejandro Pieters, Folkard Asch, Simone Graeff-Hönninger

**Affiliations:** 1 Agronomy, Institute of Crop Science, University of Hohenheim, Stuttgart, Germany; 2 Department Sustainable Agriculture and Energy Systems, University of Applied Science, Weihenstephan-Triesdorf, Germany; 3 Institute for Agricultural Science in the Tropics (Hans Rutenberg Institute), University of Hohenheim, Stuttgart, Germany

**Keywords:** cannabidiol (CBD), controlled environment agriculture, deficit irrigation, drought timing, genotype, inflorescence biomass, water use efficiency

## Abstract

Drought stress (DS) can restrict plant growth while potentially enhancing secondary metabolism through adaptive physiological responses. However, its effect on cannabinoid production remains uncertain, with previous studies reporting inconsistent findings. This study investigated how DS intensity and timing affect inflorescence biomass, cannabidiol (CBD) concentration, and CBD yield in two chemotype III (CBD-dominant; high-CBD, low-THC) medicinal cannabis genotypes differing in architecture and growth habit.: Two DS intensities were imposed during flowering relative to a well-watered control held at 60–80% of container capacity (CC) with a daily drip supply of 50–300 mL per plant. Under moderate DS, the substrate was allowed to dry to 20–30% CC before a daily replenishment of 50–150 mL was applied to prevent wilting; this cycle was repeated at three flowering stages (early, 46 days after planting [DAP]; mid, 60 DAP; late, 79 DAP). Under severe DS, water was withheld completely until the onset of wilting, as a single event at one of these three stages. Controlled water deficit elicited clear physiological responses, including reductions in relative water content, osmolality, transpiration, photosynthesis, and stomatal conductance, demonstrating effective perception of and adaptation to water limitation. Despite these responses, drought stress had only limited effects on inflorescence biomass and cannabinoid yield. Severe DS consistently impaired plant performance and provided no agronomic advantage. Although it sharply raised leaf-level water-use efficiency (WUEi), this reflected a short-term survival response achieved at the expense of carbon assimilation. Moderate DS reduced water consumption without negatively affecting biomass or CBD yield. Notably, genotype exerted a stronger influence on productivity and cannabinoid accumulation than the imposed irrigation regime. These findings indicate that severe drought stress is not a suitable strategy for enhancing cannabis production. Instead, moderate water deficit represents a practical approach to improving water-use efficiency while maintaining yield under controlled-environment cultivation. Overall, optimizing genotype selection appears to be a more effective strategy for maximizing productivity and cannabinoid yield than applying drought stress to stimulate secondary metabolism.

## 1 Introduction

Cannabis (*Cannabis sativa* L.) is an ancient crop cultivated over millennia for its medicinal, nutritional, and functional values (Clarke and Merlin, 2016). In the last decade, the world has witnessed a revival of cannabis cultivation as countries are turning away from prohibition to decriminalize and regulate cannabis products. Different from hemp (cannabis varieties for industrial purposes), medicinal cannabis refers to pharmaceutical-grade *Cannabis sativa* L. cultivars, generally cultivated under environmentally controlled conditions in indoor facilities (Zheng, 2022) to be standardized for medical use. They are classified according to their cannabinoid profile into chemotype I (THC-dominant), chemotype II (balanced THC/CBD), and chemotype III (CBD-dominant).However, indoor systems used for medicinal cannabis cultivation are characterized by high energy consumption and remarkable carbon and water footprints (Dillis et al., 2020; Mills, 2012; Zheng et al., 2021). Additionally, improper waste disposal and water pollution are further environmental impact pathways associated with indoor cannabis cultivation (Wartenberg et al., 2021). Although some studies indicate a high water demand for cannabis cultivation, most have analyzed the impacts of outdoor cultivation of industrial hemp and medicinal cannabis on local watersheds (Bauer et al., 2015; Butsic and Brenner, 2016; Dillis et al., 2020; Zheng et al., 2021). Nonetheless, concrete studies on indoor cannabis transpiration and water footprint remain absent from the literature, as these results are highly variable depending on genotype and cultivation system (Campbell et al., 2019).

Cannabis inflorescence biomass and cannabinoid yields can be modulated through agronomic management techniques, such as genotype selection (Rafiq et al., 2024), pruning techniques (Crispim Massuela et al., 2022; Danziger and Bernstein, 2021a; Folina et al., 2020), nutrition (Bernstein et al., 2019; Da Cunha Leme Filho et al., 2020; Drotleff, 2022; Gorelick and Bernstein, 2017; Massuela et al., 2023; Westmoreland and Bugbee, 2022), lighting (Danziger and Bernstein, 2021b; Eaves et al., 2020; Reichel et al., 2022), substrate composition (Barrett et al., 2016; Burgel et al., 2020; Schober et al., 2023), and drought stress (DS) intensity and timing (Caplan et al., 2019; Duong et al., 2023; Morgan et al., 2024). DS is one of the most significant abiotic stresses that reduce plant growth and yields due to stomatal closure and metabolic limitations (Akula and Ravishankar, 2011; Park et al., 2022; Rahnama et al., 2024). Water deficit is directly related to a variety of cellular processes, including transpiration, carbon assimilation, carbohydrate transport and metabolism, signal transduction mechanisms, and secondary metabolite biosynthesis, transport, and catabolism (Gao et al., 2018; Park et al., 2022; Saloner and Bernstein, 2021; Song et al., 2023). On the other hand, DS can also be a major stimulant of secondary metabolite production in plants through protective and adaptive responses (eustress) (Kleinwächter and Selmar, 2015). However, evidence that such an eustress effect operates in cannabis remains preliminary and is largely extrapolated from other essential-oil and medicinal crops (Sharma et al., 2025).

The medicinal and commercial value of cannabis rests largely on its cannabinoids, a class of secondary metabolites synthesized and stored in the glandular trichomes of the female inflorescence. Of the more than one hundred cannabinoids identified in the plant, cannabidiol (CBD) and Δ^9^-tetrahydrocannabinol (THC) are the most therapeutically and economically important. Cannabinoid biosynthesis is itself a protective branch of secondary metabolism, and is therefore mechanistically tied to plant water status. A mild water deficit is hypothesized to act as a eustress that upregulates protective secondary metabolites through reactive oxygen species signaling (Kleinwächter and Selmar, 2015), while the same deficit constrains photosynthesis, carbon assimilation and inflorescence growth (Khabbazi et al., 2026). Because cannabinoid yield is the product of concentration and biomass, water availability and phytochemical accumulation (cannabinoid development) are inseparable.

For medicinal cannabis, the influence of controlled DS on increasing yields and cannabinoid production is a matter of discourse among researchers and cultivators. Whether this represents an unsupported industry claim or a sound agricultural practice is barely addressed in the literature. The role of DS in modulating cannabinoid yield remains unclear, and results reported in the literature are contradictory (Caplan et al., 2019; Duong et al., 2023). With climate change projected to exacerbate freshwater scarcity (Transnational Institute, 2022), a selection of genotypes exhibiting improved water-use efficiency, along with agronomic management techniques that reduce water consumption, will be key to maintaining cropping productivity.

Previous studies investigating the effects of drought stress (DS) on medicinal cannabis have reported contrasting results, suggesting that the response depends strongly on genotype and stress management. Caplan et al. (2019) found that severe DS applied late in flowering increased THCA (Δ9-tetrahydrocannabinolic acid) and CBDA (cannabidiolic acid) concentrations by 12% and 13%, respectively, while increasing inflorescence dry weight by 30%, indicating that carefully timed water limitation may enhance cannabinoid production. In contrast, Duong et al. (2023) reported no significant effect of severe drought on CBD (cannabidiol) concentration in two high-CBD genotypes but observed substantial reductions in inflorescence biomass, resulting in lower CBD yields. Likewise, Morgan et al. (2024) showed that severe drought decreased both inflorescence yield and cannabinoid concentrations, whereas moderate water deficit reduced irrigation requirements without significantly affecting yield or cannabinoid content.

Beyond the effects of a single severe drought event, further research is needed on repeated exposure of cannabis plants to DS, as this may lead to acclimation responses, such as drought responses that reduce water status and photosynthesis, and eventually trigger an increase in secondary metabolites. It is worth noting that an extended drought period or recurrence may be necessary to induce this response in plants that have already undergone acclimation (Caplan et al., 2019). Finally, the effect of DS on indoor cultivated high-CBD cannabis plants is still not well understood and, thus, of great importance for the sustainability of production systems (Caplan et al., 2019).

These contrasting findings likely reflect differences in genotype, chemotype, and the severity, duration, and timing of the applied DS treatments. Consequently, the interactions among these factors remain poorly understood, and no consensus has been reached regarding the use of DS as an agronomic strategy to improve cannabinoid production. Furthermore, little is known about the effects of repeated drought cycles, particularly in indoor-grown high-CBD cultivars, despite their potential relevance for improving water-use efficiency and the sustainability of medicinal cannabis production systems. Therefore, a clear understanding of how these factors interact is crucial for reconciling the existing literature and developing reliable agronomic protocols.

Given the conflicting findings and methodological variations in the existing literature, this study was designed to evaluate the effects of two critical factors: DS intensity (moderate vs. severe) and its timing across three distinct stages of flowering for two high-CBD genotypes with different plant architectures and growth behaviors. While previous research has often focused on single stress events or intensities, our approach provides a more granular understanding of the plant’s response dynamics. Furthermore, this study integrates changes at the substrate level (decline in container water capacity) and physiological responses at the leaf level (relative water content, osmolality) with agronomic outcomes, tracing the pathway from stress responses to final cannabinoid yield. We hypothesized that a controlled, moderate application of DS throughout flowering reduces water consumption and increases water use efficiency without reducing CBD concentration or yield, whereas severe DS reduces inflorescence biomass and, consequently, CBD yield. To test this, the objectives of this study were (i) to investigate the effect of severe DS at three different phenological stages during flowering and (ii) to evaluate the application of a recurrent moderate drought treatment in comparison to the severe single-stress treatments and the well-watered control.

## 2 Materials and methods

**Figure 1:**
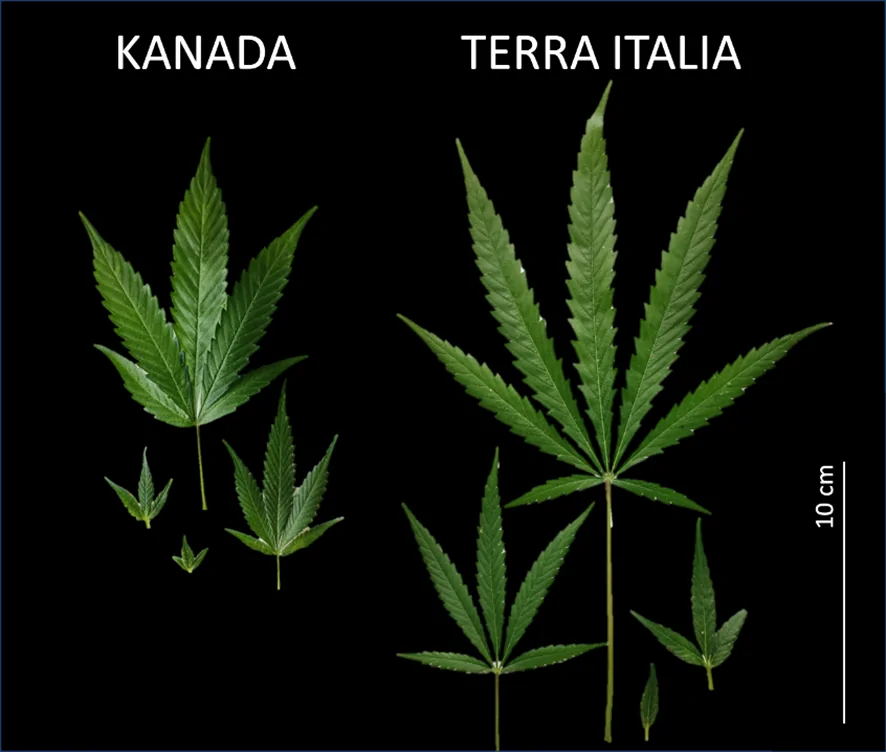
Exemplary leaves representing multiple plants of the genotypes KANADA (left) and TERRA ITALIA (right) at the final harvest date (79 days after planting), showing morphological differences among genotypes.

**Figure 2:**
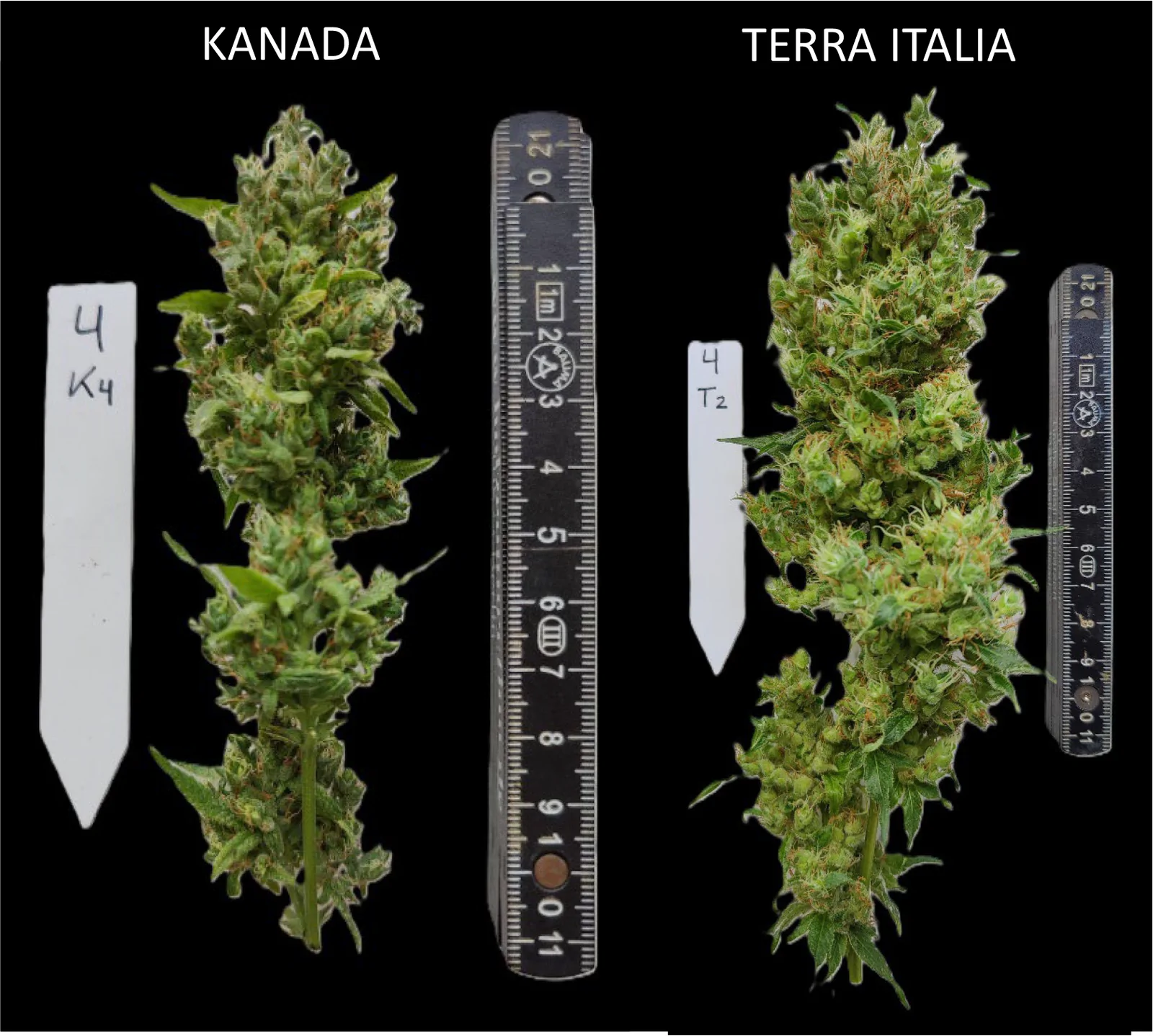
Exemplary inflorescence structure of the genotypes KANADA (left) and TERRA ITALIA (right) at the final harvest date (79 days after planting).

An indoor experiment was performed at the University of Hohenheim (Stuttgart, Germany) between March and June 2022. Plants from two high-CBD (chemotype III, CBD ~16%, THC > 0,3%) medicinal genotypes with different architectural and growth characteristics were used in this study, namely Kanada (KAN), provided by AiFame (Wald-Schönengrund, Switzerland), and Terra Italia (TIT), provided by Female Seeds (Amsterdam, Netherlands). ‘KAN’ is a compact, earlier-maturing genotype (daylength requirement ~14 h) that establishes most of its leaf area during the vegetative phase, whereas ‘TIT’ is a taller, more vigorous, and later-maturing genotype (daylength requirement 12–13 h) with a larger leaf area. Exemplary leaf and inflorescence morphology are presented in **Figures 1** and **2**. Plants were grown in a cultivation room inside the greenhouse complex of the Phytotechnikum (University of Hohenheim). Air temperature and humidity were constantly monitored. During the cultivation period, the daily mean air temperatures ranged from 18.2 to 39.0 °C, and relative humidity ranged from 11.4 to 76.1%, comparable to standard greenhouse conditions. Plants were cultivated under ambient atmospheric CO_2_ concentrations of 420–430 ppm CO_2_ (µmol mol^−^¹).

### 2.1 Planting material and cultivation methods

Plants were generated by cloning standardized stock mother plants. Clones (cuttings) were derived from apical tips of upper branches of a single mother plant for each genotype, then dipped into 1% Rhizopon AA powder (Rhizopon, Rijndijk, Netherlands) and transferred into EazyPlugs (3.5 cm × 3.5 cm × 3.5 cm) (Goirle, Netherlands). These cuttings were cultivated in a nursery greenhouse, with humidity maintained above 80% and adequate ventilation for proper air circulation. After 19 days, rooted cuttings were transplanted into 4.5 L square pots with 915 ± 1 g of a peat-based, commercially available substrate - Substrate-5 (recipe 446, without fertilizer and 10% perlite) (Klasmann-Deilmann GmbH, Geeste, Germany). Pots were topped with 300 ± 1 g of gravel to reduce water loss due to evaporation. The day of transplanting was considered the beginning of the experiment, defined as the initial day after planting (DAP), i.e., 0 DAP. Pots were placed in three rows, each with ten pots on horticultural tables (1.0 m × 2.5 m), resulting in a density of 12 plants m^-^². In total, 44 plants per genotype (88 plants) were grown on three tables. Treatments were randomly assigned to pots within each table, with individual tables serving as replicates. In the first and third tables, 29 plants were allocated. The remaining 30 plants were put on the second table. In each replicate, a row-column design was used to allocate combinations of genotype, treatment, and replicate further described in section 2.6.

By the vegetative period, the photoperiod length was 18 h, provided by ceramic metal halide lamps (CHD Agro 400W; DH Licht GmbH, Wülfrath, Germany). The total vegetative duration after planting was 15 days. During the generative period, the photoperiod was 12 h and lasted for 64 days. Plants were checked regularly for the appearance of male flowers to avoid pollination. The final harvest occurred after nine weeks of flowering (79 DAP), as reported in previous experiments with the same genotype (Crispim Massuela et al., 2022). Pests were controlled biologically using auxiliary predatory insect populations (spp. *Phytoseiulus persimilis, Amblyseius Californicus, Orius Majusculus*, and *Aphidoletes aphidimyza*) provided weekly by the company Sautter & Stepper (Ammerbuch, Germany).

An organic fertilizer solution was prepared by mixing Phytogreen^®^ Bio NPK 5-2–5 without molasses (Phytosolution, Freyburg, Germany), Carbon Eco (Phytosolution, Freyburg, Germany), and Epsom salt. The concentrations [mg L^-1^] of macronutrients in the prepared nutrient solutions were 240 N, 96 P, 240 K, 74 Ca, and 18.5 Mg (corresponding to approximately 17.1 mM N, 3.1 mM P, 6.1 mM K, 1.9 mM Ca, and 0.8 mM Mg), following Massuela et al. (2023). Fertilization occurred twice a week and did not occur during the drought. The total fertilizer amount per plant during the total cultivation period was 816 mg N, 326.4 mg P, 816 mg K, 251.6 mg Ca, and 62.9 mg for KAN and 1128 mg N, 451.2 mg P, 1128 mg K, 347.8 mg Ca, and 87 mg for TIT. Phosphorus was supplied at a level higher than in a standard full-strength Hoagland solution, reflecting the formulation of the organic fertilizer used and the elevated P demand of cannabis during inflorescence development.

A drip irrigation system with a controller was mounted on the pots to provide a constant water supply of 50–300 mL per day, depending on the plants’ growth stage and environmental conditions. Neither fertilizer solution nor irrigation water leached through the pots. All plants received the same amount of fertilizer solution (mL plant^-1^). Water levels in pots were measured by a randomized weighing routine and maintained between 60 and 80% of container capacity (CC); this water status in the substrate is considered the control.

### 2.2 Drought treatments

**Figure 3:**
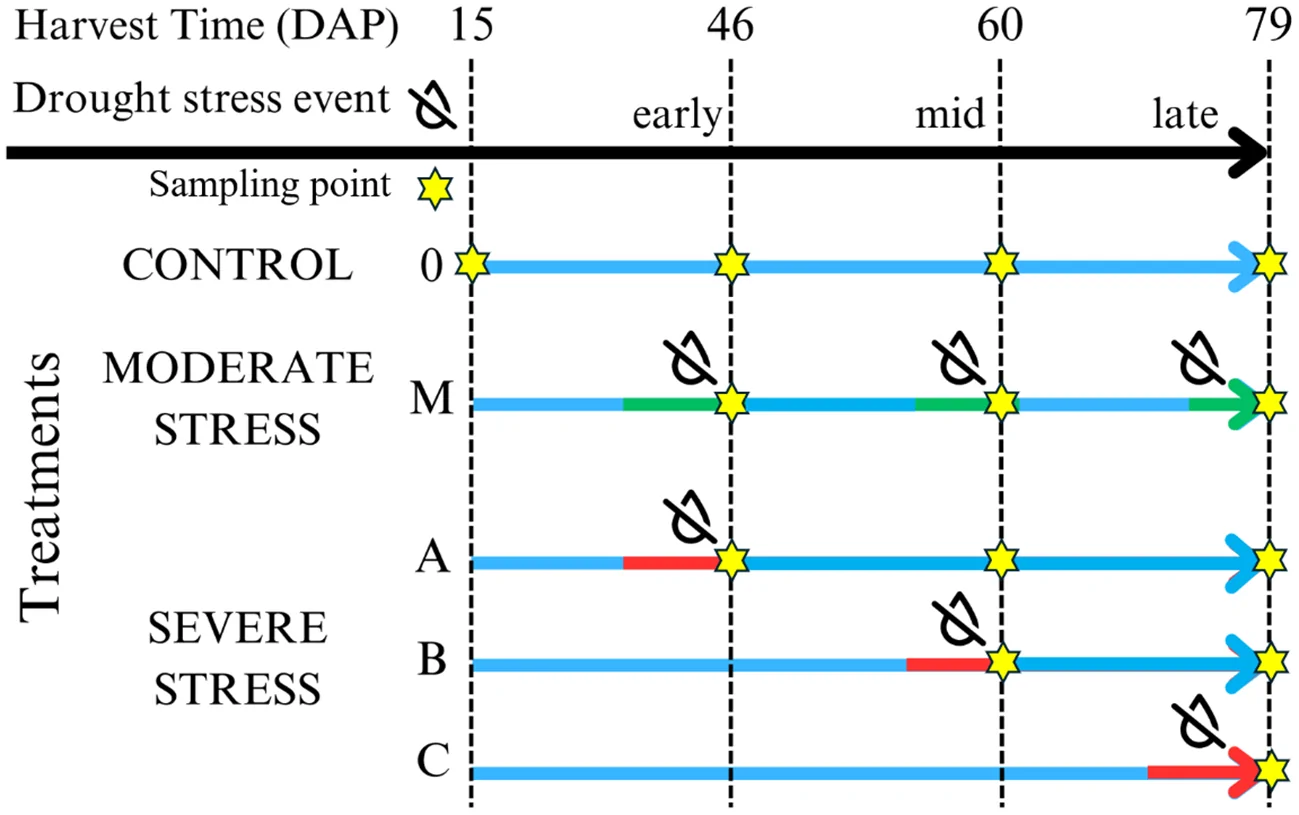
Graphical representation of treatments across harvest dates (DAP) and drought stress events (early, mid, late). The black water drop symbol indicates which treatments were exposed to the drought event and when. The yellow star indicates which treatments were harvested at a particular harvest time.

During the flowering stage, two drought treatments were applied. I) moderate stress (M), in which the substrate was allowed to dry until reaching the plateau (around 20 – 30% CC), plants then received a daily replenishment of water (50–150 mL) to prevent leaf wilting; and II) severe DS, in which plants would not receive water until wilting or the end of the drought event. The severe DS was split into three treatments occurring at different phenological stages: A) at early flowering (46 DAP) – by the appearance of trichomes in inflorescences (4 weeks of flowering); B) at mid flowering (60 DAP) – ripening of inflorescences (6.5 weeks of flowering); and C) at late flowering (79 DAP) – by optimum harvest time (9 weeks of flowering). The phenological stages were defined in prior experiments with the same genotype (Crispim Massuela et al., 2022). For the severe stress treatments, plants were exposed to a single drought event (either by early (A), mid (B), or late (C) flowering), while moderate stress treatment (M) plants were exposed to all three drought events. Between consecutive drought events, M plants were returned to and maintained at control conditions (60–80% CC), so that the three moderate-stress cycles were separated by the intervals between the early (46 DAP), mid (60 DAP), and late (79 DAP) phenological stages. Plants from DS treatments were kept at control conditions before and after the drought events. Additionally, control plants (0) were cultivated without DS. A graphical representation of treatments, showing DS events and harvest dates, is displayed in **Figure 3**.

### 2.3 Plant sampling

During the cultivation period, four destructive harvest events were conducted. Harvests were conducted at the end of the vegetative stage (15 DAP) and three times during the flowering stage at 46, 60, and 79 DAP (**Figure 3**). At the first harvest event, all plants were treated equally, as drought treatments were applied afterward. Therefore, only control plants were harvested. At the second harvest, only plants treated in the first drought event (drought treatments A and M) differed from the control (0). Thus, three drought-by-harvest combinations exist. At the third harvest, there are four additional combinations (A, B, M, 0). At the final harvest, all drought treatments existed and differed from each other. Therefore, a total of 13 combinations were harvested.

At the first three harvest events (15, 46, and 60 DAP), three plants per treatment (one plant per combination and replicate) were cut at the base and separated into the fractions: stems, leaves (15, 46, 60 DAP), and inflorescences (46, 60 DAP). The inflorescences were hand harvested and additionally separated into three fractions based on plant height (top, mid, and low) to account for inner-plant variation in total CBD concentration and CBD yield, as demonstrated by Crispim Massuela et al. (2022). Inflorescences were hand trimmed to remove sugar leaves. At the last harvest, four plants per treatment were harvested. The latter requires that these five combinations be replicated twice within one of the three replicates during the experiment’s randomization. Stem and leaf samples were oven-dried at 60 °C for 48 h. The inflorescences were air-dried in an air-circulating chamber at temperatures ranging from 20 to 28 °C and relative humidity between 30 and 60% until a target moisture content of 12% was reached. All dried samples were weighed to determine dry matter. For cannabinoid analysis, inflorescence samples were ground to a homogeneous powder using an ultra-centrifugal mill (Type ZM 200; Retsch, Haan, Germany). The residual moisture of each inflorescence sample was measured with a moisture analyzer (DBS 60-3; Kern and Sohn GmbH, Balingen, Germany).

### 2.4 Measurement of stress levels

During the experiment, the largest fully developed fan leaf was used to measure photosynthesis, transpiration, stomatal conductance, and water deficit (wilting point, relative water content, and osmolality). This leaf will be referred to in this work as ‘major leaf’. The leaf was selected at the initiation of flowering and marked on every plant.

Leaf area was measured with an LI-3100 Area Meter (LI-COR, Lincoln, USA); specific leaf area (SLA) (cm² g^-1^) was calculated as the ratio of the leaf area (cm²) divided by leaf dry matter (g) for each plant.

#### 2.4.1 Photosynthetic rate and SPAD

The photosynthetic rate, transpiration rate, and stomatal conductance were measured with the gas-exchange system GFS-3000 (Heinz Walz GmbH, Effeltrich, Germany). Environmental conditions in the measurement cuvette were set to 30 °C leaf temperature, 50 ± 5% relative humidity, and 405 ± 5 ppm CO_2_ concentration. Measurements were made at 2000 µmol m^-2^ s^-1^ PAR (Amax).

At each harvest, SPAD readings were taken from leaves at three different canopy positions based on plant height (low, mid, top). The selected leaves were: for the low position, the oldest non-senescent fan leaf; for the mid position, the largest and most developed fan leaf; and for the top position, the youngest fully developed fan leaf. The average of four measurements was recorded for each leaf using a SPAD-502 (Konica Minolta, Chiyoda, Japan), and the plant average was calculated as the mean across the three canopy positions.

#### 2.4.2 Water deficit in substrate

Before the experiment, the maximum container capacity (analogous to ‘field capacity’, used for field experiments) was calculated by saturating the substrate, measuring the substrate weight after 48 hours, and deducting the weight of the dry substrate, which was measured after the substrate was dried at 70 °C for 48 hours (Casaroli and Jong van Lier, 2008).

During each drought event, all plants were watered to 60-80% of container capacity. Then, drip irrigators were removed from the plants that would undergo drought, and substrate water levels started to reduce over time. All pots were weighed twice daily in the morning and late afternoon to monitor the drying curves of the substrate; once the plateau was reached (around 20-30% of container capacity), leaves began to wilt. The plants in the moderate stress treatment (M) received daily water replenishment (50–150 ml) to prevent leaf wilting. The plants subjected to severe drought treatments (A–C) did not receive any water replenishment. The stress event was finalized once the major leaf wilted by more than 50° relative to the main stem, as suggested by Caplan et al. (2019) as a drought-stress indicator for cannabis plants. Each drought event lasted approximately 140 hours, that applies to all drought events and for both moderate and severe stress treatments. Once the drought was finalized, the substrate was rehydrated to control conditions, and plants were harvested after 4 hours of rehydration.

#### 2.4.3 Water deficit in the leaves

For leaf water deficit measurements, the major leaf of each plant was collected at the end of each drought event, before rehydration, for biomass harvest. Four leaf discs (2.4 cm²) from the central leaflet were cut and used to calculate relative water content (RWC) following the method of González and González-Vilar (2001). The remaining major leaf material was used to measure osmolality (OD) by pressing it against a fine plastic tube to extract leaf sap, which was then stored at -20 °C until analysis with an Osmomat 3000 (Gonotec, Germany). The Osmomat 3000 uses the freezing-point depression method to measure osmolality (mOsm/kg H_2_O). The instrument determines how much the sample’s freezing point is lowered relative to pure water. Because freezing-point depression is proportional to the number of dissolved osmotically active particles, the instrument directly calculates the sample’s osmolality.

### 2.5 Cannabinoid analysis

The total CBD concentration of inflorescences was analyzed by high-performance liquid chromatography (HPLC and calculated following the methods and equations presented in Massuela et al. (2023). Cannabinoid extraction was performed using 100 ± 10 mg of dried inflorescences in 100 mL of a 90% methanol/10% chloroform (v/v) (9 + 1) mixture in an ultrasonic bath for 30 min at 40 °C. After cooling down, the solution was filtered through syringe filters Polytetrafluorethylen (PTFE), 0.45 µm (Macherey-Nagel GmbH & Co. KG, Germany) into HPLC vials and injected into the HPLC system (1290 Infinity II LC System, Agilent, Santa Clara, USA) equipped with an autosampler, a quaternary pump, as well as a diode-array spectrophotometer (DAD) at the detection wavelength of 230 nm. The chromatographic separation was carried out on a Nucleosil 120–3 C8 column (125 mm × 4 mm i.d., 3.0 µm) with an EC 4/3 Nucleosil 120–3 C8 guard column (Macherey-Nagel, Oensingen, Switzerland). The mobile phase was a mixture of HPLC-grade methanol (solvent A) and 0.1% acetic acid in HPLC-grade distilled H_2_O (solvent B; Sigma-Aldrich, Saint Louis, MO, USA) at a constant flow rate of 0.7 mL min-1 with gradient elution mode. The injection volume was 10 µL, and the total run time comprised 27 min. The integration of targeted peaks was performed using cannabinoid reference standards for CBD (C-045) and CBDA (C-144) (Sigma-Aldrich, Germany), and data analysis was carried out using the ChemStation software for LC Rev. B.04.03-SP2 (Agilent, Santa Clara, USA). Calibration curves were generated from diluted standard solutions, with coefficients of determination of 1.0 for both CBD and CBDA. The limit of detection for CBD and CBDA was 0.0015%.

Total CBD concentration (%) was calculated as a weighted sum of CBD (%) and CBDA (%) in each inflorescence sample. The multiplication by the factor 0.877 accounts for the differences in molar mass between the acid and neutral forms of the cannabinoid, as one molecule of CO_2_ is lost during decarboxylation (Equation 1):

(1) $${\text{CBD}}_{\text{Total}}=\text{CBD}+\text{CBDA}\times 0.877$$

To correctly evaluate cannabinoid production capacity, the calculated yield of total CBD (mg plant^−1^) must be taken into consideration. Yield was calculated considering inflorescence fresh weight, the residual moisture of the analytical sample and the total CBD concentration, using Equation (2). The conversion factor 0.2 represents the average dry matter concentration of fresh inflorescences and was applied to calculate the yield at the moisture of the analytical sample. The residual moisture was the weight proportion of water in the dried samples. The total CBD yield was calculated for each sample as follows (Equation 2):

(2) $${\text{CBDyield}}_{\text{Total}}={\text{CBD}}_{\text{Total}}\cdot \text{Inflorescence fresh weight}\times (0.2\;\times {\left(1-\text{residual moisture}\right)}^{-1})$$

### 2.6 Statistical design

Data from the destructive measures taken per plant were analyzed using the following mixed model:


$$y_{hijklm}=\mu +b_{h}+r_{hl}+c_{hm}+\tau_{i}+\rho_{j}+\delta_{jk}+{\left(\tau \rho \right)}_{ij}+{\left(\tau \delta \right)}_{ijk}+e_{hijklm}$$

where $y_{hijklm}$ is the observation in row *l* and column *m* of replicate *h* treated with genotype *i* at harvest *j* and drought-harvest-combination *k* nested within *j*, μ is the intercept, $b_{h}$, $r_{hl}$, and $c_{hm}$ are the fixed effects of replicate *h* and the random effects of row *l* and column *m* nested within replicate *h*. The terms $\tau_{i}$, $\rho_{j}$, and $\delta_{jk}$ are the fixed effects of genotype *i*, harvest event *j* and harvest-by-drought treatment combination *k*, ${\left(\tau \rho \right)}_{ij}$ and ${\left(\tau \delta \right)}_{ijk}$ are the fixed interaction effects of the corresponding main effects and $e_{hijklm}$ is the error effect of $y_{hijklm}$ with genotype-specific variance. The latter was selected because the heterogeneous variance model showed a better fit. The model fit was measured via AIC (Wolfinger, 1993). An analogous model was fitted to repeated-measures data from the final harvest only. In these cases, the effect of harvest event was replaced by canopy position. Additionally, a first-order autoregressive variance-covariance structure with heterogeneous variances for canopy positions was assumed. As only data from six to seven plants per replicate exist, random row and column effects could not be fitted. In all cases, residuals were visually checked for homogeneity of variances (despite the model’s heterogeneity) and normality. For CBD yield, data were logarithmically transformed prior to analysis to fulfill requirements. Afterward, Fisher´s LSD test was performed for significant effects. Results from multiple comparisons are presented via letter display. For CBD yield, mean estimates were back-transformed for presentation purposes only. Standard errors were back-transformed using the Delta method.

For drying curves, a non-linear function based on the Page Model equation (Simpson et al., 2017) was fitted to observations for each plant. The following model for the metric time variable (*t*) was assumed, where *t* is the time (hours) of the drought event:


$$\eta_{hijklm}=e^{-k(t_{hijklm}^{2})}$$

where $\eta_{hijklm}$ is the expected value of the plant grown in replicate *h*, row *i*, column *m*, and treated with genotype *I* and drought-by-harvest combination *jk*. The variables *k* and η are the parameters of the curve. Afterward, parameter estimates across plants were analyzed with the model (1). To avoid convergence problems, row and column effects were dropped from the model. Based on the significance of genotype and harvest-by-drought treatment combinations, mean parameters were estimated and plugged into the model equation for the curve. Finally, raw data of t and CC were plotted in scatter plots, and a simple regression with its 95% confidence limits was shown for visual representation only.

## 3 Results and discussion

The results and discussion are presented in order of causation to follow the pathway of drought stress (DS) responses, from substrate- and leaf-level physiology, through the effects on plant growth and inflorescence biomass, and ultimately on cannabinoid (CBD) concentration and yield.

**Figure 4:**
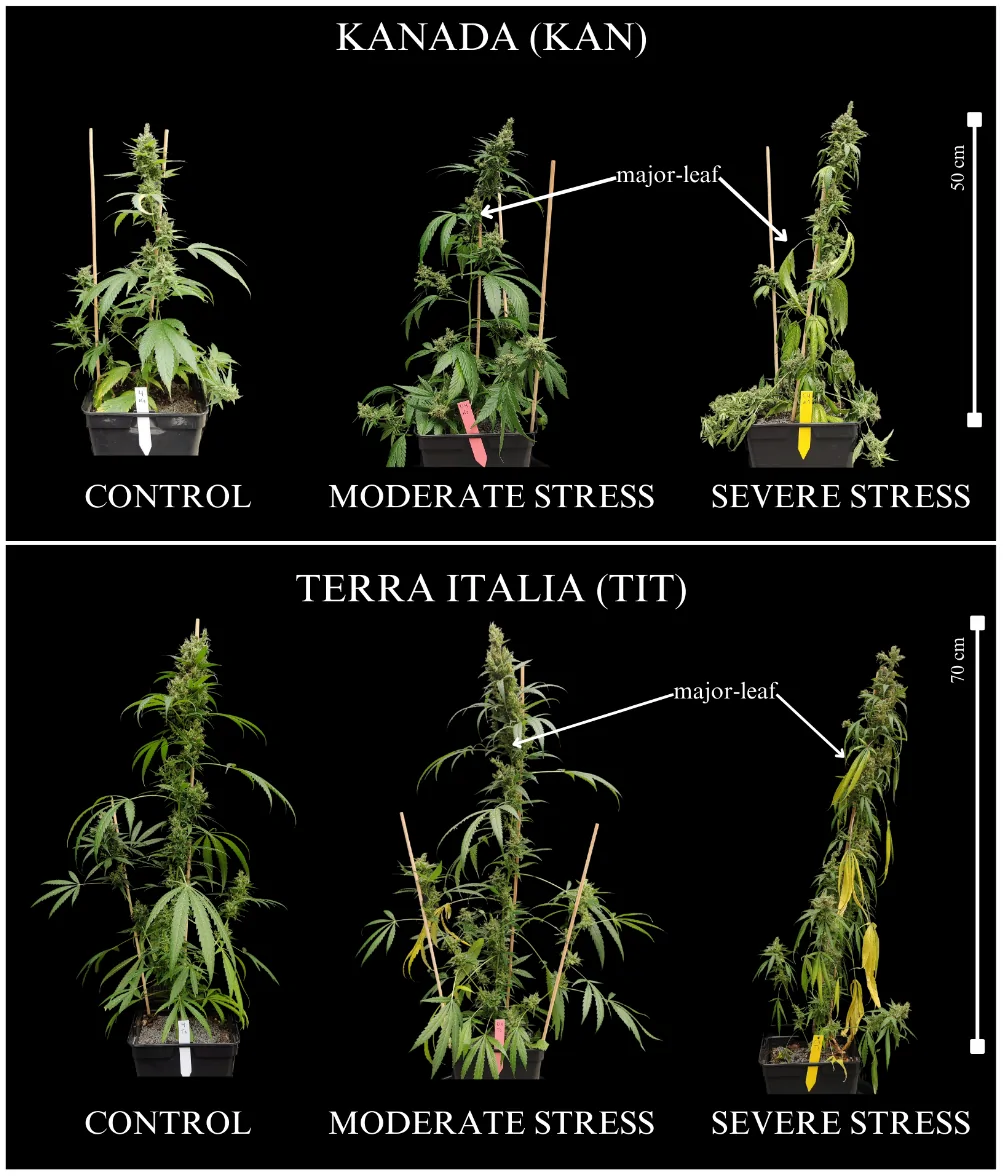
Exemplary plants from the genotypes KANADA (above) and TERRA ITALIA (below) at the final harvest date (79 DAP), with the applied treatments: Control (left), Moderate stress (center), and Severe stress (right). Arrows indicate exemplary positions of the major leaf.

The major factors related to DS investigated were genotype-dependent. Therefore, all results will be presented separately for the KAN and TIT genotypes. Particular stress-coping strategies can be observed when plants experience drought. TIT plants were taller and had greater leaf area; they exhibited higher, faster water consumption and thus wilted more quickly than KAN plants. On the other hand, the shorter KAN plants consumed water more slowly and wilted later than TIT plants. **Figure 4** visualizes plants of different treatments. By the end of the stress period, unlike plants under severe stress, plants under moderate stress did not exhibit wilting of the major leaf.

### 3.1 Drought at the substrate level

**Figure 5:**
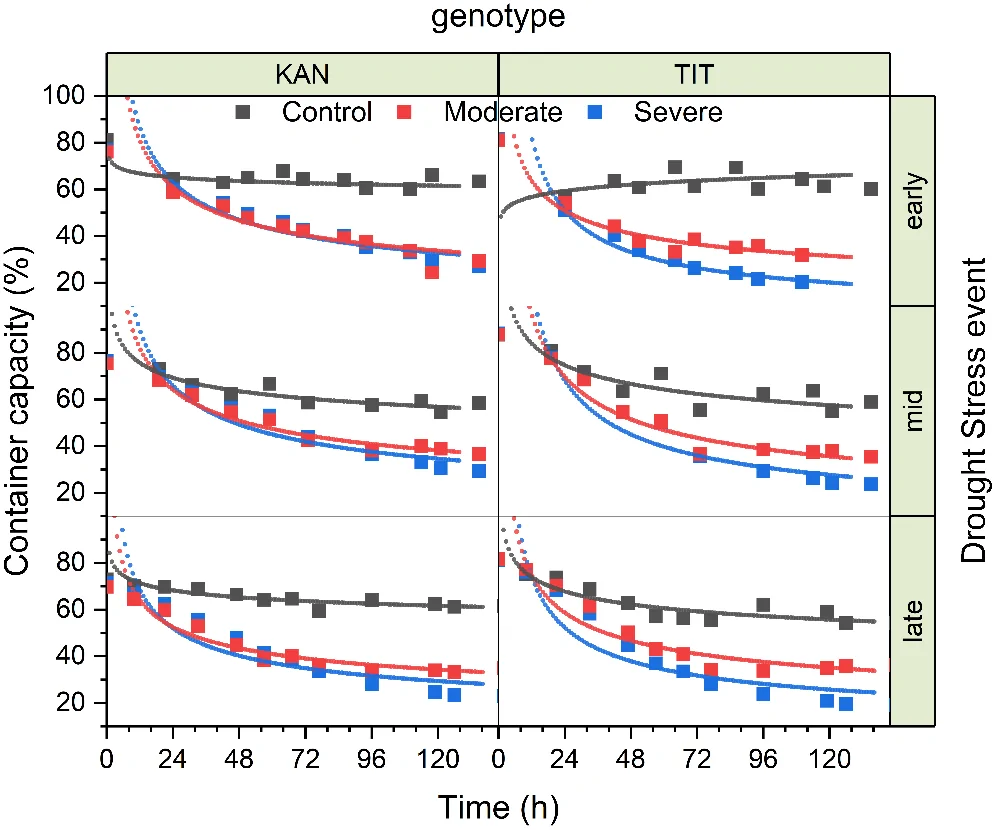
Reduction in CC (Container capacity, %) over time and under drought stress treatments during the three drought stress events (early, mid, late) for the genotypes KAN and TIT. The plotted curves are fitted regressions using equation (2).

At the substrate level, as water was consumed by plants, the weight of pots decreased over time. Single-pot weights were measured to account for plant water status, represented by CC (%). In **Figure 5**, the change in CC over time is shown for the severe and moderate drought stress during the three drought events (early, mid, late) and the control for both genotypes (KAN, TIT). The CC of control treatments was maintained between 60 and 80%, whereas the moderate stress treatment reduced the CC to a plateau of around 40% (wilting point). The severe stress treatments dried further until major leaf wilting occurred at around 20-30% CC. The pairwise comparisons of coefficients fitted using equation (2) showed significant differences among the drying curves of the control, moderate, and severe stress treatments for both genotypes and all drought events.

#### 3.1.1 Drought at the leaf level

Relative water content (RWC) is a measure of leaf water content and can indicate water stress in plants. A lower RWC indicates a higher water deficit in the major leaf tissue, which characterizes DS at the top of the plant (after progressive wilting of leaves from the bottom to the top). For both genotypes, RWC was significantly lower in the DS treatment compared with the control (**Table 1**), except for KAN at 60 DAP (mid stress) and TIT at 46 DAP (early stress). Unlike severe stress treatments, moderate stress treatments did not show significantly lower RWC values, indicating that leaves were not wilting and that the major leaf had a similar water status to control treatments. Across DS events, RWC values in severely stressed plants were 1.9 to 23.2% lower than in control plants for KAN and 5.2 to 66.5% lower for TIT plants. Similarly, Asadi et al. (2022) reported RWC of 53% for severe, 64% for mild, and 77% for well-watered treatments.

**Table 1:** Relative water content (RWC) for control and drought stress treatments at three different drought events (early, mid, and late flowering) for both genotypes, Kanada (KAN) and Terra Italia (TIT).

Genotype	Treatment	RWC (%)		
		Drought stress event (DAP)		
		early (46)	mid (60)	late (79)
KAN	control	79 ± 1.8^1^ **a**	92.3 ± 1.6 **a**	89.8 ± 1.6 **a**
	moderate	70.3 ± 1.8 **b**	94.2 ± 1.6 **a**	94.7 ± 1.6 **a**
	severe – early	60.7 ± 1.8 **c**		
	severe – mid		90.5 ± 1.6 **a**	
	severe – late			83.1 ± 1.6 **b**
TIT	control	77.8 ± 2.2 **a**	91.3 ± 1.3 **a**	89.4 ± 4.5 **a**
	moderate	81.0 ± 2.2 **a**	92.7 ± 1.3 **a**	87.5 ± 4.5 **a**
	severe – early	73.8 ± 2.2 **a**		
	severe – mid		85.5 ± 1.3 **b**	
	severe – late			59.5 ± 4.5 **b**

^1^Means with different letters are significantly different from each other according to Fisher’s LSD test with **α = 0.05**.

The osmolality (OS) indicates the concentration of osmotically active solutes in leaf tissue and is a sign of water status in the cell tissue. The higher the OS, the higher the concentration of osmotically active solutes in the major leaf, indicating a lower water content relative to the amount of solutes. During each drought event, the highest OS was observed in the severe drought treatment for both cultivars (**Table 2**). Moderate stress did not show significantly different OS from the control, indicating no water deficit in the major leaf.

**Table 2:** Osmolality (OS) of the genotypes Kanada (KAN) and Terra Italia (TIT) under control and drought stress treatments across three drought events (early, mid, and late flowering).

Genotype	Treatment	Osmolality (mOsm kg^-1^ H_2_O)		
		DAP (day after planting)		
		46	60	79
KAN	control	553 ± 32^1^ **a**	593 ± 48 **a**	620 ± 69 **b**
	moderate	607 ± 32 **a**	571 ± 48 **a**	731 ± 69 **ab**
	severe – early	642 ± 32 **a**		
	severe – bottom		730 ± 59 **a**	
	severe – late			863 ± 69 **a**
TIT	control	578 ± 9 **b**	584 ± 25 **b**	643 ± 37 **b**
	moderate	588 ± 9 **b**	553 ± 25 **b**	667 ± 37 **b**
	severe – early	746 ± 9 **a**		
	severe – bottom		713 ± 25 **a**	
	severe – late			937 ± 37 **a**

^1^Means with different letters are significantly different from each other according to Fisher’s LSD test with **α = 0.05**.

Asadi et al. (2022) reported significant differences in physiological and metabolic stress responses across DS treatments and cannabis ecotypes native to Iran. Unfortunately, the authors did not report biomass and cannabinoid data. However, it was reported that severe and mild DS treatments significantly decreased total chlorophyll and relative water content in leaves while significantly increasing membrane leakage. Concerning metabolites, plants under severe and mild stress showed a significant decrease in carotenoid concentrations while presenting significantly higher levels of proline, catalase, and guaiacol peroxidase. Those biomarkers are related to stress-tolerance responses in plants, aimed at maintaining cell turgor and osmotic balance and preventing oxidative stress induced by reactive oxygen species (Gebicka and Krych-Madej, 2019; Hayat et al., 2012). Consistent with the researchers’ results, both KAN and TIT plants demonstrated stress-coping mechanisms to maintain cell turgor and osmotic balance, as evidenced by increased leaf OP despite reductions in RWC.

### 3.2 Effect on transpiration, photosynthesis, and stomatal conductance

**Figure 6:**
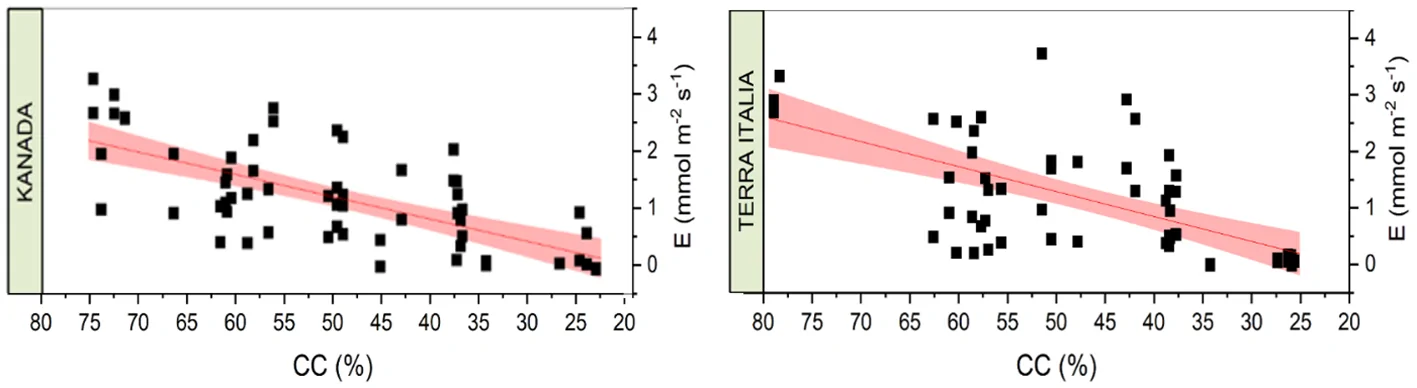
Transpiration rate (E) by Container capacity (CC) for the genotypes KAN (left) and TIT (right). The plotted lines are linear regressions, and the shaded areas are 95% confidence bands.

Drought stress reduces photosynthesis due to stomata closure and metabolic limitations. Primary metabolism is limited by water status in leaf cells, as indicated by lower RWC (**Table 1**) and higher OS (**Table 2**). Plants therefore had limitations in carbon assimilation and, thus, in biomass accumulation. With the reduction of water in the substrate - here translated by the container capacity (CC) –transpiration also declined (**Figure 6**). Transpiration values ranged from 2.0 to 3.3 mmol m^-2^ s^-1^ in well-watered plants to nearly zero under severe stress. Plants under moderate stress exhibited high variability in transpiration rates (0.2–2.8 mmol m^-2^ s^-1^).

**Figure 7:**
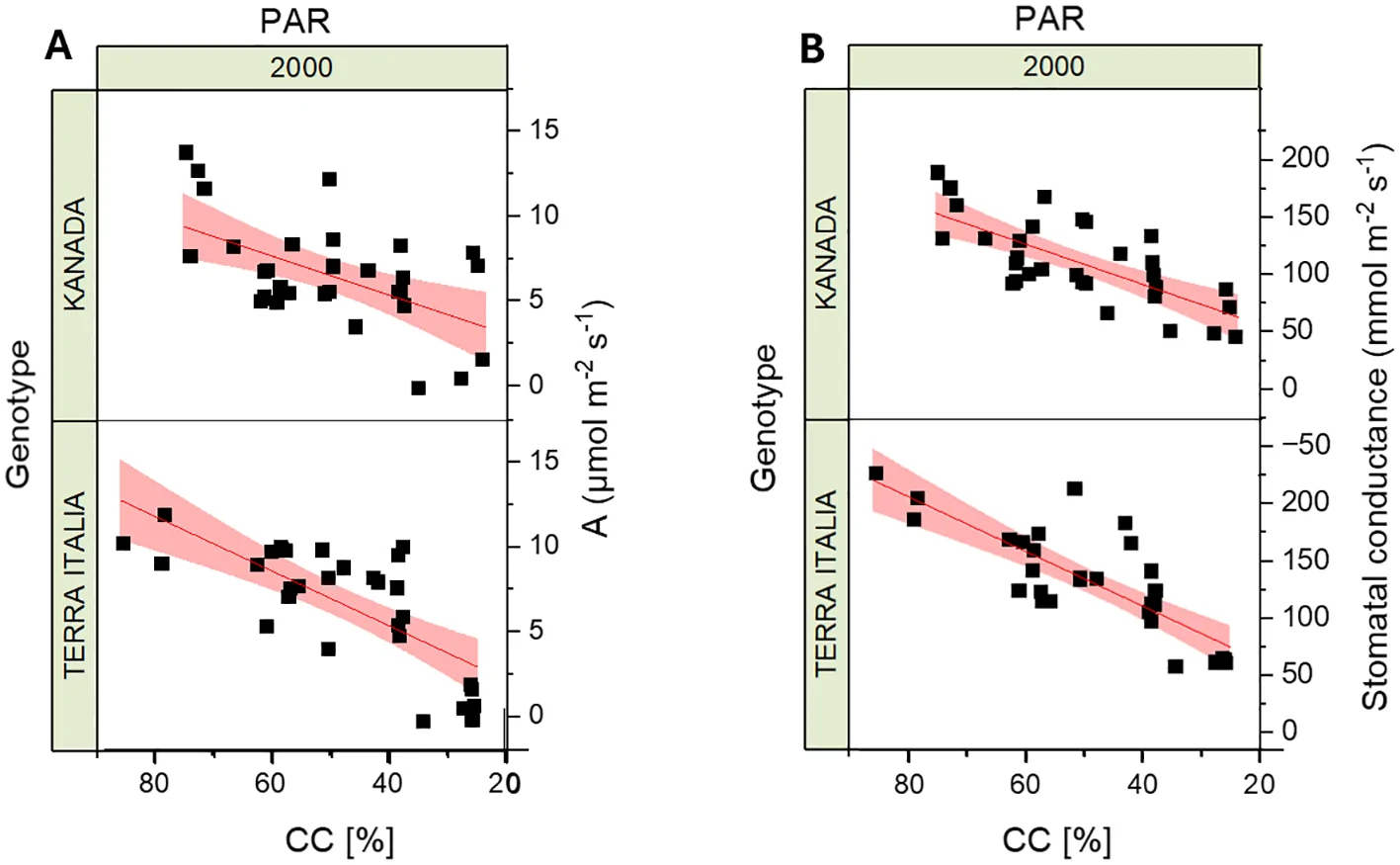
Photosynthetic rate **(A)** and Stomatal conductance **(B)** as a function of Container Capacity (CC) for the genotypes KAN and TIT at PAR levels of 2000. The plotted lines are linear regressions, and the shaded areas are 95% confidence bands.

Similar trends were observed for photosynthetic rates (A) and stomatal conductance, which decreased in parallel with reduced container capacity. Photosynthetic rates were reduced by about 5–10 times. This pattern was observed in measurements of 2000 PAR for both genotypes (**Figure 7**). In control conditions, Amax (2000 PAR) ranged from 5.0 to 12.7 µmol m^-2^ s^-1^ for KAN and 7.1 to 11.9 µmol m^-2^ s^-1^ for TIT. On the other hand, stomatal conductance values ranged from 30 to 150 mmol m^-2^ s^-1^ for both genotypes and at both PAR levels. Generally, below 30% of CC, little to no photosynthesis or transpiration occurred. The reduction in stomatal conductance under DS is consistent with findings by Rahnama et al. (2010), who demonstrated that stomatal closure is a rapid response to osmotic stress, serving as an early mechanism to limit water loss and maintain osmotic balance. In general, KAN plants are reported to be sturdier under drought conditions, suggesting possible tolerance to drought stress. Values for photosynthetic rate under well-watered conditions are slightly lower than the reported range (13–25 µmol m^-2^ s^-1^), while values for stomatal conductance are consistent with previously reported values ranging from 50 to 150 mmol m^-2^ s^-1^ (Chandra et al., 2008, 2011).

The heterogeneous responses of plants to similar stress levels (same CC water status) observed in transpiration, photosynthetic rate, and stomatal conductance confirm that stress reactions may vary among individuals (Duong et al., 2023). Even when using clones from the same stock plant, we cannot guarantee homogeneity of plant responses, as these depend on several environmental factors and the microclimate around the plants. Therefore, applying DS would lead to less standardization among plants and individual inflorescences within a batch, which is detrimental to compliance with Good Manufacturing Practice (GMP) guidelines regarding maximum variation in cannabinoid concentration within a batch.

**Figure 8:**
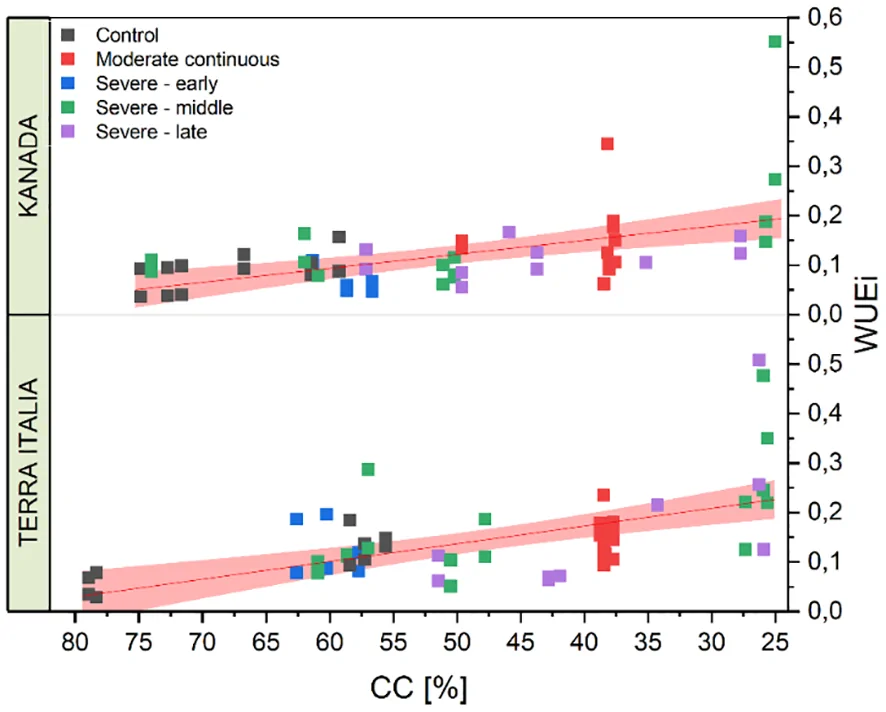
Intrinsic Water Use Efficiency (WUEi) by Container Capacity (CC) for the genotypes KAN and TIT. The plotted lines are linear regressions, and the shaded areas are 95% confidence bands.

**Figure 8** shows the intrinsic water use efficiency (WUEi) for the drought stress treatments (in colors), calculated by dividing the photosynthetic rate by stomatal conductance, which can be interpreted as the leaf-level response of water use efficiency (WUE). Plants under moderate stress did not show significant differences in WUEi compared to control plants. In contrast, plants under severe drought treatments showed WUEi values that were double or even triple those of control plants (CC < 30%). However, the increase in WUEi under severe stress reflects a short-term survival mechanism that prioritizes hydraulic safety and water conservation at the expense of carbon assimilation (Sharma et al., 2025). The associated reduction in stomatal conductance and photosynthesis, while improving instantaneous efficiency, ultimately limits biomass accumulation (Al-Salman et al., 2024; Peters et al., 2018). The ranges for plants under well-watered conditions (0.05-0.15) are consistent with the reported literature (Herppich et al., 2020).

Both genotypes showed similar leaf-level responses to DS. Despite reductions in RWC, increased OS suggested osmotic adjustment to maintain osmotic balance, whereas transpiration, photosynthetic activity, and stomatal conductance were reduced during DS events. The reported results confirm that DS occurred, and DS treatments yielded significant differences compared with the control for the aforementioned stress parameters (**Tables 1**, **2**) and (**Figures 6**–**8**). Under DS conditions, plants increased water-use efficiency at the leaf level (**Figure 8**); this coping mechanism for drought stress has also been reported by other authors (Morgan et al., 2024).

### 3.3 Plant growth and inflorescence biomass

As plant growth and biomass responses to DS were predominantly genotype-dependent, the results for ‘KAN’ and ‘TIT’ are analyzed and discussed separately. Consequently, this section focuses on the factors that demonstrated significant effects. An ANOVA summary of the most relevant sources of variation is presented in **Table 3**; factors and interactions that were not statistically significant (e.g., stem diameter, height, SPAD) are omitted for clarity (**Table 3**).

**Table 3:** ANOVA table for the sources of variation: Days after planting (DAP), treatment (trt), Genotype (geno), and their interactions. DAP is nested within the factor (trt) to compare treatments at each harvest event.

Source of variation	Leaf Area	Inflorescence Dry Matter	CBD %	CBD yield
DAP	**0.0371**	**<.0001**	**<.0001**	**<.0001**
trt(DAP)	**<.0001**	0.1497	0.4377	0.0663
geno	**<.0001**	**<.0001**	**<.0001**	**<.0001**
geno*DAP	0.2963	**<.0001**	**<.0001**	**<.0001**
geno*trt(DAP)	**0.0004**	0.1405	0.8448	0.1274

p-values correspond to the F-test for differences between levels of the corresponding factor. The p-values with statistical significance (*p* < 0.05) were highlighted in bold.

**Figure 9:**
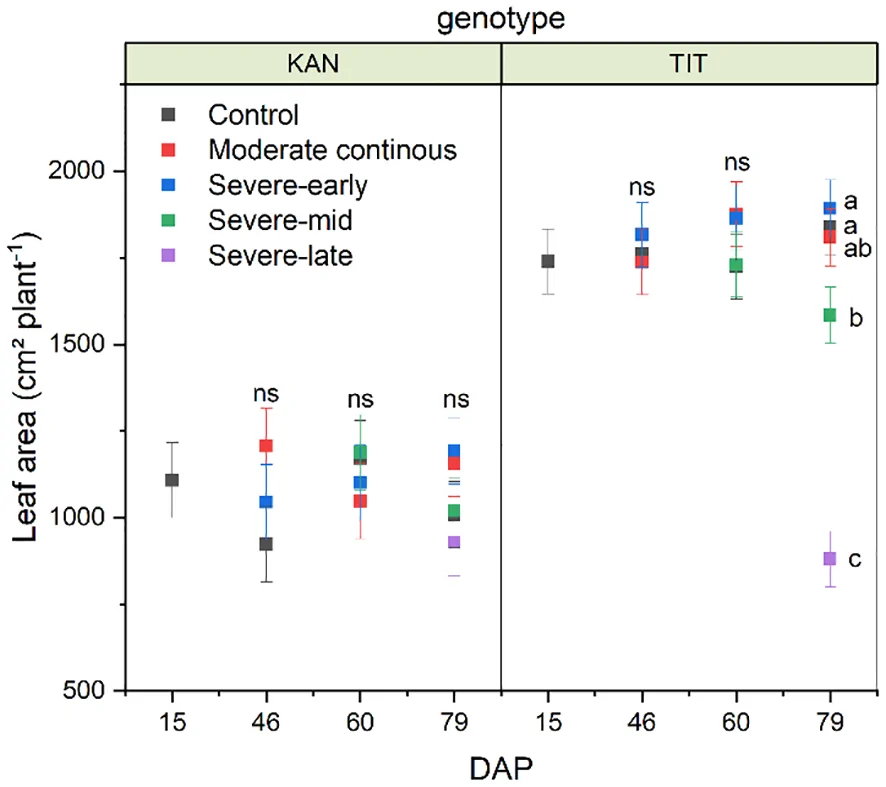
Leaf area (cm² plant^-1^) by days after planting (DAP) for the genotypes KAN and TIT. Means with different letters are significantly different from each other according to Fisher’s LSD test with α = 0.05. Means with ns are not significantly different from each other according to global F-test.

Leaves were the plant organ most responsive to DS treatments. Leaf area (LA) was significantly reduced by water deficit, with a clear genotypic divergence in coping strategy. The genotype ‘KAN’ exhibited a conservative strategy. Having already established most of its leaf area (1108 cm² plant^−^¹) by the end of the vegetative stage (15 DAP), it maintained its LA throughout flowering (923–1207 cm² plant^−^¹), with no significant reduction from DS treatments (**Figure 9**). This suggests a reallocation of resources rather than a loss of photosynthetic capacity. In contrast, the larger ‘TIT’ plants employed an avoidance strategy, shedding leaves in response to severe stress. This led to pronounced leaf senescence, particularly during the late DS event. At 79 DAP, plants in the severe-late treatment had less than half the LA (879.6 cm² plant^−^¹) of the control plants (1838.7 cm² plant^−^¹), a reduction significantly greater than that observed in the severe-mid treatment (1584.6 cm² plant^−^¹) (**Figure 9**). This indicates that ‘TIT’ abscises larger fan leaves to minimize water loss, a response that intensified with later stress application.

Cannabis plants reallocate water and nutrients from leaves and stems to inflorescences in response to stressful events as a survival strategy (Massuela et al., 2023). Either by reducing transpiration (**Figure 6**) or by reducing leaf area (**Figure 9**), plants could maintain water and nutrients in reduced ‘sugar’ leaves and continue providing assimilates for inflorescence growth, thereby maintaining a steady accumulation of inflorescence biomass over time (**Table 4**).

**Table 4:** Inflorescence dry matter (g plant^-1^) for the drought stress treatments across three different drought events (early, mid and late flowering) for the genotypes Kanada (KAN) and Terra Italia (TIT).

Genotype	Treatment	Inflorescences DM (g plant^-1^)			
		DAP (days after planting)			
		15	46	60	79
KAN	control	1.8 ± 0.5	3.3 ± 0.5	5.1 ± 0.5	5.5 ± 0.4 **b**
	moderate		4.0 ± 0.5	3.8 ± 0.5	7.1 ± 0.4 **a**
	severe – early		3.5 ± 0.5	3.8 ± 0.5	7.2 ± 0.4 **a**
	severe – mid			5.0 ± 0.5	5.3 ± 0.4 **b**
	severe – late				7.4 ± 0.4 **a**
TIT	control	2.6 ± 0.6	5.4 ± 0.6	9.3 ± 0.6	13.3 ± 0.5
	moderate		5.3 ± 0.6	8.2 ± 0.6	12.3 ± 0.5
	severe – early		5.2 ± 0.6	8.8 ± 0.6	12.9 ± 0.5
	severe – mid			8.7 ± 0.6	13.1 ± 0.5
	severe – late				13.4 ± 0.5

Means with different letters are significantly different from each other according to Fisher’s LSD test with α = 0.05. Means without letters are not significantly different from each other according to global F-test.

The final inflorescence DM at harvest (79 DAP) was primarily determined by genotype, with ‘TIT’ producing approximately twice the biomass of ‘KAN’ (**Table 4**). Within each genotype, DS treatments had a limited effect.

In contrast, for the higher-yielding ‘TIT’, none of the DS treatments significantly altered final inflorescence biomass relative to the well-watered control. These results demonstrate that while a genotypic capacity for higher yield is a fixed trait, the marginal yield increase observed in ‘KAN’ under specific DS regimes was not a consistent response across genotypes.

### 3.4 CBD concentration and yield

In KAN plants of the control treatment, total CBD concentration increased from 2.9% to a maximum of 4.5% by mid-flowering (60 DAP), followed by a reduction to 3.4% by late flowering (**Table 5**). For the TIT genotype, total CBD concentrations in control plants increased from 3.4% to a maximum of 9.3% by mid-flowering (60 DAP) and remained at that level until the final harvest (79 DAP). The increases in CBD concentration under moderate stress at the final harvest (14.7% for ‘KAN’ and 2.2% for ‘TIT’) were not statistically significant. Ultimately, neither CBD concentration nor yield was significantly affected by DS, as the final yield was predominantly determined by the strong genotypic difference in inflorescence biomass.

**Table 5:** Total CBD concentration (%) for the drought stress treatments across three different drought events (early, mid and late flowering) for the genotypes Kanada (KAN) and Terra Italia (TIT).

Genotype	Treatment	Total CBD (%)			
		DAP (days after planting)			
		15	46	60	79
KAN	control	2.9 ± 0.2	3.0 ± 0.2	4.5 ± 0.2	3.4 ± 0.2
	moderate		3.7 ± 0.2	4.3 ± 0.2	3.9 ± 0.2
	severe – early		3.1 ± 0.2	4.0 ± 0.2	4.0 ± 0.2
	severe – mid			4.1 ± 0.2	3.5 ± 0.2
	severe – late				3.9 ± 0.2
TIT	control	3.4 ± 0.5	4.5 ± 0.5	9.3 ± 0.5	9.1 ± 0.4
	moderate		4.5 ± 0.5	9.7 ± 0.5	9.3 ± 0.4
	severe – early		4.2 ± 0.5	8.4 ± 0.5	8.9 ± 0.4
	severe – mid			9.6 ± 0.5	8.9 ± 0.4
	severe – late				9.2 ± 0.4

For KAN, the maximum CBD concentration occurred by mid-flowering (60 DAP) and then declined by the last harvest event (90 DAP) (**Table 5**). This behavior supports the hypothesis of maximum cannabinoid production capacity during mid-flowering, while the reduced values at late flowering (**Table 5**) can be attributed to a dilution effect due to biomass growth (**Table 4**). The same trend was observed in prior experiments with the same genotype (Crispim Massuela et al., 2022). This trend was not observed in TIT plants, as the CBD concentration reached a maximum by mid-flowering and remained at that level through the final harvest. This suggests that the plant may still accumulate total CBD yield; therefore, nine weeks of flowering may not be the optimum harvest time for TIT, as demonstrated for KAN in Crispim Massuela et al. (2022).

As a key agronomic metric, CBD yield is the product of inflorescence biomass and CBD concentration. Consequently, the non-significant effect of DS treatments on CBD concentration (**Table 5**) meant that final yield trends mirrored those of inflorescence dry matter (DM) (**Table 4**). Although the main treatment effect (trt(DAP)) was not significant (p = 0.0663; **Table 3**), a significant genotype-by-treatment interaction was observed for ‘KAN’ (**Table 6**), driven primarily by variations in its inflorescence DM. Reflecting the combined trends of its yield components, the genotype ‘TIT’ achieved a CBD yield approximately five times greater than ‘KAN’. This superior performance, attributable to its faster growth rate, makes ‘TIT’ a more suitable genotype for indoor production systems targeting short cultivation cycles of less than 80 days.

**Table 6:** Total CBD yield (mg plant^-1^) for the drought stress treatments across three different drought events (early, mid, and late flowering) for the genotypes Kanada (KAN) and Terra Italia (TIT).

Genotype	Treatment	Total CBD yield (mg plant^-1^)			
		DAP (days after planting)			
		15	46	60	79
KAN	control	51.2 ± 6.9	103.9 ± 14.0	216.3 ± 29.2	187.4 ± 21.9 **b**
	moderate		149.1 ± 20.0	176.0 ± 23.6	273.5 ± 31.7 **a**
	severe – early		109.5 ± 14.8	147.9 ± 19.9	289.8 ± 33.7 **a**
	severe – mid			204.7 ± 27.5	180.8 ± 21.2 **b**
	severe – late				272.0 ± 31.9 **a**
TIT	control	96.8 ± 10.2	249.8 ± 26.5	861.1 ± 91.1	1243.6 ± 114.1
	moderate		228.8 ± 24.2	781.6 ± 82.9	1124.2 ± 104.4
	severe – early		220.7 ± 23.4	696.6 ± 74.4	1138.8 ± 105.2
	severe – mid			806.7 ± 86.4	1178.7 ± 108.2
	severe – late				1220.2 ± 112.7

Means with different letters are significantly different from each other according to Fisher’s LSD test with α = 0.05. Means without letters are not significantly different from each other according to global F-test.

The effects of DS on secondary metabolism - in this case, CBD production - of medicinal cannabis are multilayered and very complex (Kleinwächter and Selmar, 2015). As hypothesized in the introduction chapter, a controlled application of DS during flowering could increase cannabinoid biosynthesis, as reported by Caplan et al. (2019). However, due to other co-occurring impacts on osmotic regulation, transpiration, and photosynthesis, this enhancement might be offset or even overcompensated, as reported by Duong et al. (2023) and Morgan et al. (2024) for reductions in inflorescence biomass.

A broader question is whether high-CBD medicinal cannabis possesses the evolutionary and ecological predisposition to convert drought into a metabolic gain. The capacity of a species to redirect water-deficit signaling toward enhanced secondary metabolism depends on whether such stress-responsive pathways were selected for during its evolutionary history; in plants from environments where a given stress was never a recurrent selective pressure, the corresponding pathways may be only weakly developed, yielding variable and poorly reproducible responses (Sharma et al., 2025). The avoidance strategies observed here—leaf abscission and severe deformation of the major fan leaves under severe stress, while younger tissues remained comparatively unaffected—are consistent with a plant that prioritizes survival and water conservation rather than one that channels drought signaling into trichome metabolism. This may help explain why drought did not enhance cannabinoid production in either genotype and suggests that other elicitors (for example, light spectrum, mechanical or oxidative cues) might be more effective than water deficit for stimulating secondary metabolism in this crop. In this work, the observed reductions in photosynthesis and stomatal conductance in the major fan leaves (**Figure 7**) did not translate into significant limitations in inflorescence biomass accumulation (**Table 4**). We hypothesize that this discrepancy arises from source-sink dynamics within the plant during flowering. The physiological measurements were conducted on older fan leaves, whereas the newly formed reduced leaves (sugar leaves) within the inflorescences likely constitute the primary source of assimilates for inflorescence growth (Reichel et al., 2022). These sugar leaves appeared resilient to the applied drought stress, showing no wilting even under severe treatments, suggesting that the plant prioritizes water and resource allocation to these reproductive structures. Consequently, metabolic activity in the inflorescences may have been maintained, preventing biomass penalties. At the mechanistic level, the eustress hypothesis posits that water deficit increases the accumulation of reactive oxygen species (ROS), which can act as signals to upregulate the biosynthesis of protective secondary metabolites such as cannabinoids and terpenoids (Sharma et al., 2025). In the present study, however, the stress imposed on the major fan leaves did not translate into a measurable increase in CBD biosynthesis, most likely because the inflorescence sink—where cannabinoids are synthesized and stored in glandular trichomes—remained hydrated and metabolically active. This decoupling between leaf-level stress signaling and inflorescence metabolism offers a parsimonious explanation for the absence of a cannabinoid response and is consistent with recent reports that drought effects on cannabinoid concentration are weak and strongly genotype-dependent (Cappello Fusaro et al., 2025; Pena et al., 2025) (**Table 4**). While this study provides insights into two high-CBD genotypes, future research should expand to a wider genetic pool, including THC-dominant and balanced varieties, to fully characterize genotypic responses to DS. Furthermore, investigations into the interactions between DS and other environmental factors, such as light intensity and vapor pressure deficit, are needed. A critical next step is also to evaluate the impact of DS on the full spectrum of secondary metabolites, including terpenoids and flavonoids, building on recent work that resolved terpene profiles in CBD-dominant cannabis under water stress (Cappello Fusaro et al., 2025), to understand its comprehensive effect on the phytochemical profile and overall product quality (Campbell et al., 2019; Caplan et al., 2019; Gorelick and Bernstein, 2017).

This study highlights a critical knowledge gap, as the physiology of cannabis sugar leaves under drought has not yet been reported in the literature. Future research should directly investigate the photosynthetic response of sugar leaves to longer drought exposures. Furthermore, building on findings that moderate stress can maintain yields while increasing WUEi (**Figure 8**), with significantly reduced water use (Morgan et al., 2024), it would be valuable to explore stress acclimation by comparing recurrent moderate stress against constant low-level deficit irrigation to optimize water use efficiency without compromising yield (Caplan et al., 2019).

Furthermore, it should be noted that this study was limited to two high-CBD genotypes grown under controlled indoor conditions over a single flowering cycle. The physiological measurements were primarily conducted on mature fan leaves, potentially overlooking the role of sugar leaves as key sources of assimilates during flowering. Future research should expand the genetic scope to include THC-dominant and balanced chemotypes, directly assess sugar leaf physiology under stress, and evaluate multi-cycle stress applications to confirm stability of the observed responses.

## 4 Conclusion

This study assessed how the intensity and timing of drought stress (DS) during flowering affect inflorescence biomass, cannabidiol (CBD) concentration, and CBD yield in two architecturally contrasting high-CBD (chemotype III) medicinal cannabis genotypes. Across all treatments, DS was reliably imposed and perceived by the plants. Physiological responses translated into only marginal effects on the agronomic endpoints, and neither inflorescence biomass, CBD concentration, nor CBD yield were significantly altered by the irrigation regime.

Severe DS was consistently detrimental and offered no benefit. It triggered leaf abscission and canopy loss, which was most pronounced in the vigorous ‘Terra Italia’ (TIT) genotype, where late-flowering stress reduced leaf area to less than half that of the control. Although it raised leaf-level water-use efficiency (WUEi) two- to threefold, this reflected a short-term hydraulic-safety response achieved at the expense of carbon assimilation rather than a genuine productivity gain. In moderate DS, relative water content and osmolality remained comparable to the well-watered control. Water consumption was lowered while fully maintaining biomass and CBD yield. Notably, DS did not stimulate cannabinoid biosynthesis in either genotype; the modest increases in CBD concentration under moderate stress were not statistically significant.

Across every yield-determining trait, genotype outweighed the imposed irrigation regime. ‘TIT’ produced approximately twice the inflorescence biomass and five times the CBD yield of the compact, earlier-maturing ‘Kanada’ (KAN), which instead reached its peak CBD concentration earlier in flowering. For producers, selecting a well-adapted, high-yielding genotype and matching harvest timing to its cannabinoid trajectory is therefore a far more decisive lever for productivity and cannabinoid output than manipulating irrigation to induce stress. We conclude that moderate deficit irrigation is a viable and sustainable means of reducing water use in indoor medicinal cannabis production without compromising yield or quality, while severe stress should be avoided. Optimizing genotype selection remains the primary route to maximizing productivity, and the interactions between deficit irrigation, genotype, and other environmental elicitors such as light spectrum and vapor pressure deficit represent a promising avenue for further improvement.

## Data Availability

The datasets used and/or analysed during the current study are available from the corresponding author on reasonable request.
